# High-Quality eHealth Websites for Information on Endometriosis: Systematic Search

**DOI:** 10.2196/48243

**Published:** 2024-01-10

**Authors:** Diksha Sirohi, Cecilia Hoi Man Ng, Niranjan Bidargaddi, Helen Slater, Melissa A Parker, Mary Louise Hull, Rebecca O'Hara

**Affiliations:** 1 Robinson Research Institute Adelaide Medical School University of Adelaide North Adelaide Australia; 2 School of Clinical Medicine Division of Obstetrics and Gynaecology University of New South Wales Sydney Australia; 3 Jean Hailes for Women's Health Melbourne Australia; 4 Digital Health College of Medicine and Public Health Flinders University Adelaide Australia; 5 Curtin School of Allied Health Curtin University Perth Australia; 6 Canberra Endometriosis Centre Centenary Hospital for Women and Children ACT Health Canberra Australia

**Keywords:** digital health, endometriosis, eHealth websites, eHealth, pelvic pain, adenomyosis

## Abstract

**Background:**

eHealth websites are increasingly being used by community members to obtain information about endometriosis. Additionally, clinicians can use these websites to enhance their understanding of the condition and refer patients to these websites. However, poor-quality information can adversely impact users. Therefore, a critical evaluation is needed to assess and recommend high-quality endometriosis websites.

**Objective:**

This study aimed to evaluate the quality and provide recommendations for high-quality endometriosis eHealth websites for the community and clinicians.

**Methods:**

PRISMA (Preferred Reporting Items for Systematic Reviews and Meta-Analyses) 2020 guidelines informed 2 Google searches of international and Australian eHealth websites. The first search string used the terms “endometriosis,” “adenomyosis,” or “pelvic pain,” whereas “Australia” was added to the second search string. Only free eHealth websites in English were included. ENLIGHT, a validated tool, was used to assess the quality across 7 domains such as usability, visual design, user engagement, content, therapeutic persuasiveness, therapeutic alliance, and general subjective evaluation. Websites with a total score of 3.5 or more were classified as “good” according to the ENLIGHT scoring system and are recommended as high-quality eHealth websites for information on endometriosis.

**Results:**

In total, 117 eHealth websites were screened, and 80 were included in the quality assessment. Four high-quality eHealth websites (ie, those that scored 3.5 or more) were identified (Endometriosis Australia Facebook Page, Endometriosis UK, National Action Plan for Endometriosis on EndoActive, and Adenomyosis by the Medical Republic). These websites provided easily understood, engaging, and accurate information. Adenomyosis by the Medical Republic can be used as a resource in clinical practice. Most eHealth websites scored well, 3.5 or more in the domains of usability (n=76, 95%), visual design (n=64, 80%), and content (n=63, 79%). However, of the 63 websites, only 25 provided references and 26 provided authorship details. Few eHealth websites scored well on user engagement (n=18, 23%), therapeutic persuasiveness (n=2, 3%), and therapeutic alliance (n=22, 28%). In total, 30 (38%) eHealth websites scored well on general subjective evaluation.

**Conclusions:**

Although geographical location can influence the search results, we identified 4 high-quality endometriosis eHealth websites that can be recommended to the endometriosis community and clinicians. To improve quality, eHealth websites must provide evidence-based information with appropriate referencing and authorship. Factors that enhance usability, visual design, user engagement, therapeutic persuasiveness, and therapeutic alliance can lead to the successful and long-term uptake of eHealth websites. User engagement, therapeutic persuasiveness, and therapeutic alliance can be strengthened by sharing lived experiences and personal stories and by cocreating meaningful content for both the community and clinicians. Reach and discoverability can be improved by leveraging search engine optimization tools.

**Trial Registration:**

PROSPERO CRD42020185475; https://www.crd.york.ac.uk/PROSPERO/display_record.php?RecordID=185475&VersionID=2124365

## Introduction

Endometriosis is a chronic condition causing pain and fertility problems in 5%-10% of natal females globally [1]. It is associated with an average of 6-8 years delay in diagnosis [2,3], which is compounded by uncertainty for health care providers over optimal management [4]. Endometriosis requires long-term therapeutic strategies and appropriate access to medical services to reduce the negative impacts on quality of life such as anxiety, depression, pain during sex, difficulty in doing household tasks, or caring for children due to chronic pelvic pain [5].

People with endometriosis commonly use eHealth [6] websites to seek information about endometriosis when their symptoms persist and are not effectively addressed in traditional health care settings [7,8]. In this context, “What is endometriosis?” was the third highest trending health-related question on Google in 2018 [8,9]. More than 400,000 Google searches on endometriosis are carried out per month in the United States alone [10]. An Australian study showed that a Google search for “endometriosis” increased by 26.4% after the announcement of the 2018 National Action Plan for Endometriosis in Australia [11]. Digital information seeking, including the use of eHealth websites, can contribute to improved health literacy [12].

Clinicians use evidence-based eHealth websites for medical education and when providing more information to patients [13,14]. Of 108 surveyed clinicians, 59% (n=64) had recommended a website to a patient [15]. However, the difficulty in determining the evidence base for eHealth websites and concerns over the quality of the content were identified as barriers to their use in clinical practice [8,13].

Incorrect and inaccurate information on eHealth websites can adversely affect people’s health [16,17]. In a systematic review by Hirsch et al [10] that included 54 eHealth websites providing information on endometriosis, over one-third did not cite authorship and almost half did not report references or sources of information. In a study that screened 25 eHealth websites providing information on dysmenorrhea (painful periods), a symptom commonly associated with endometriosis, only 28% included the name and credentials of the author [18]. The aim of this systematic review using the PRISMA (Preferred Reporting Items for Systematic Reviews and Meta-Analyses) guideline [19] was to evaluate the quality of endometriosis-related eHealth websites.

## Methods

### Search Strategy

**Figure 1:**
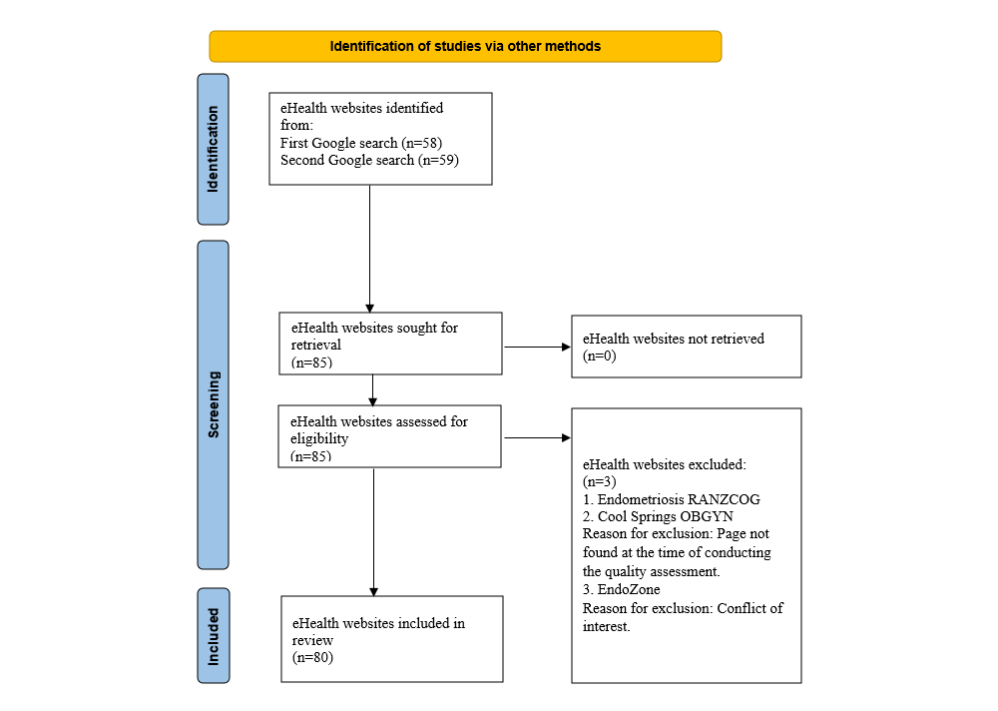
PRISMA (Preferred Reporting Items for Systematic Reviews and Meta-Analyses) 2020 flowchart.

Two Google searches were performed following PRISMA guidelines on July 27, 2020, to assess both international (.com) and Australian (.com.au) websites (Multimedia Appendix 1). The first search was conducted on Google.com using the search terms “endometriosis” OR “adenomyosis,” OR “pelvic pain.” The second search was conducted on Google.com.au and “Australia” was added to the search string (Figure 1). Google accounts for 92.26% of the global market share compared to Bing (Microsoft Corp; 2.83%), and Yahoo Search provides results generated by Bing [20,21]. Hence, we reported results based on Google search only. To minimize the impact of any previous search history, the search was conducted in “incognito” mode. The first 30 eHealth websites listed were screened as most people do not investigate beyond this number [22]. The search was later updated on August 24, 2023, to include more recent websites. Duplicate results were removed, and the eHealth websites were screened for eligibility.

### Inclusion and Exclusion Criteria

eHealth websites were included if they related to endometriosis or adenomyosis or pelvic pain, were written in English, and were free. eHealth websites that did not meet the inclusion criteria were excluded (Textbox 1).

**Inclusion and exclusion criteria for including eHealth websites on endometriosis.**

**Inclusion criteria**

- eHealth websites that relate to endometriosis, adenomyosis, and pelvic pain in women
- Free eHealth websites (no associated cost)
- eHealth websites written in the English language

**Exclusion criteria**

- eHealth websites that did not relate to endometriosis, adenomyosis, and pelvic pain in women
- eHealth websites that require a payment or subscription to access them
- eHealth websites written in a language other than English

### Data Extraction

Descriptive data were manually extracted by 1 researcher (DS) after reading the initial description and purpose of each eHealth website. These data were collated in an Excel spreadsheet (Microsoft Corp), under the following categories: (1) eHealth website name, (2) hyperlink, (3) developer, (4) funder, (5) intended purpose, (6) target audience, (7) category, (8) country of origin, and (9) last updated (Multimedia Appendix 2).

### Quality Assessment

The ENLIGHT quality assessment tool [23] was used to evaluate all included eHealth websites. The ENLIGHT tool assesses seven criteria: (1) usability, (2) visual design, (3) user engagement, (4) content, (5) therapeutic persuasiveness, (6) therapeutic alliance, and (7) general subjective evaluation (Table 1). Each ENLIGHT quality assessment criterion is scored using a rating scale of 1-5 (very poor to very good and not applicable) [23].

**Table 1:** Description of the ENLIGHT quality assessment criteria, objectives, and factors assessed [23].

Quality assessment criteria	Objective	Factors assessed
Usability	Assesses the ease of learning how to use an eHealth website and the ease of using it appropriately	Navigation Learnability Ease of use
Visual design	Assesses the look and feel of the eHealth website and the visual quality of the graphical user interface	Aesthetics Layout Size
User engagement	Assesses the extent to which the eHealth website’s design attracts users to use it.	Content presentation Interactive Not irritating Targeted or tailored or personalized reports Captivating
Content	Assesses the content provided or learned while using the eHealth website	Evidence-based content Quality of information provided Complete and concise Clarity about the program’s purpose
Therapeutic persuasiveness	Assesses the extent to which the eHealth website is designed to encourage users to make positive behavior changes or to maintain positive aspects of their life	Call to action Load reduction of activities Therapeutic rationale and pathway Rewards Real data-driven or adaptive content Ongoing feedback Expectations and relevance
Therapeutic alliance	Assesses the ability of the eHealth website to create an alliance with the user in order to effect a beneficial change.	Basic acceptance and support Positive therapeutic expectations Relatability
General subjective evaluation of the program’s potential	Examines the eHealth website’s general potential to benefit its target audience based on the rater’s subjective evaluation	Appropriate features to meet the clinical aim Right mix of ability and motivation I like the program

The eHealth websites were reviewed in 2 stages. Initially, 1 researcher (DS) reviewed all included eHealth websites. Then, the eHealth websites were divided and independently reviewed by another member of the team (RO, MLH, NB, HS, MAP, and CHMN). The ENLIGHT scores of each eHealth website were collated in an Excel spreadsheet.

Discrepancies in ratings (any deviation greater than 1 rating unit) were resolved by discussion between pairs of reviewers. If evaluation differences were not resolved, a third independent assessor was consulted. After a detailed assessment, the average of the 2 reviewers’ (DS and RO or MLH or NB or HS or MAP or CHMN) ratings was used to calculate a score for each of the 7 domains (Multimedia Appendix 3).

### High-Quality eHealth Websites

A total score for each eHealth website was calculated according to the ENLIGHT formula [24] (Multimedia Appendix 3). eHealth websites with a total score of ≥3.5 are classified as “good” according to the ENLIGHT scoring system [24] and are recommended as high-quality eHealth websites for information on endometriosis. Interrater reliability was described using an intraclass correlation coefficient, which is estimated from a 2-way mixed effects model using an absolute definition of agreement [25].

### Ethical Considerations

An ethics approval was not required for this study because this was a systematic review of eHealth websites and did not involve the recruitment of participants.

## Results

### Overview

A total of 117 eHealth websites were returned in the search, 58 from the first Google search (International) and 59 from the second Google search (Australian). Thirty-two eHealth websites were duplicates (duplicate websites identified in the international and Australian search), leaving 85 that were screened for this systematic review (Figure 1). Two eHealth websites were excluded, as they were not related to the topic. There was a conflict of interest in assessing 1 website (EndoZone) [26] since the authors were responsible for its development. Two eHealth websites—Royal Australian and New Zealand College of Obstetricians and Gynaecologists [27] and Cool Springs OBGYN [28]—could not be included in the final review, as the link was no longer available. A total of 80 eHealth websites were included in the final assessment and analysis (Multimedia Appendix 2).

There were discrepancies between the first and second reviewers (DS and RO or MLH or NB, HS, MAP or CHMN) in 7% of the ratings (316 variances across 4480 total ratings). All discrepancies were resolved without needing a third reviewer. The intraclass correlation coefficient was 0.61 (95% CI 0.45-0.73), indicating that interrater reliability was moderate [25].

### Characteristics of eHealth Websites

Of the 80 eHealth websites, 44 (55%) belonged to Australian organizations, while 36 (45%) belonged to international organizations. The majority of the eHealth websites (n=49, 61%) provided education to the community on natal female pain (eg, period pain or conditions that cause pelvic pain in women), 25 (31%) were business pages of private organizations, 5 (6%) were related to endometriosis research, and 1 (1%) was a media release of a study at a university. Of the 49 eHealth websites that provided community education, 12 also provided support features to the endometriosis community. These included resources (eg, booklets, webinars), links to support group networks, and social media platforms for digital engagement with the endometriosis community (Multimedia Appendix 2). Of the 80 eHealth websites included in this study, none required payment or a subscription fee to access content.

### Target Users

The majority of the eHealth websites (n=70, 88%) were designed for use by the endometriosis community. Five (6%) eHealth websites provided information or education to health care providers and 5 (6%) provided information for researchers.

### Quality of eHealth Websites

#### Overview

The eHealth websites were evaluated using the ENLIGHT quality assessment criteria [23]. Table 2 presents the top 4 eHealth websites (ie, those with a total score of ≥3.5) according to the ENLIGHT scoring formula [24] and are recommended as high-quality eHealth websites for information on endometriosis.

**Table 2:** Top 4 eHealth websites according to the ENLIGHT scoring system.

eHealth website	Usability score	Visual design score	User engagement score	Content score	Therapeutic persuasiveness score	Therapeutic alliance score	General subjective evaluation score	Total score
Endometriosis Australia Facebook Page [29]	4.50	4.67	4.40	4.00	4.21	4.50	4.00	4.24
Understanding endometriosis—Endometriosis UK [30]	5.00	4.00	4.30	4.38	3.57	4.33	4.33	3.92
Adenomyosis: The poor cousin of endometriosis—The Medical Republic^a^ [31]	4.67	4.50	4.00	5.00	3.07	3.50	3.83	3.63
National Action Plan for Endometriosis—EndoActive [32]	4.50	4.17	2.00	5.00	3.36	4.67	4.50	3.53

^a^High-quality eHealth website for clinicians.

#### Usability

The majority of eHealth websites (n=76, 95%) scored well (≥3.5) for usability (Multimedia Appendix 3). These eHealth websites were characterized by smooth, nearly frictionless navigation. They had an intuitive interface that was easy to learn and straightforward to use. Examples of websites that scored well for usability included the Endometriosis Australia Facebook Page [29], Endometriosis UK [30], and the Endometriosis page on the Jean Hailes for Women’s Health website [33].

Of the 76 eHealth websites that scored well for usability, 42 (55%) provided education on natal female pain to the endometriosis community, 23 (30%) were business pages of private organizations that encouraged users to book appointments, 5 (7%) eHealth websites provided education to health care providers, 5 (7%) were eHealth websites related to endometriosis research, and 1 (1%) was a media release article.

#### Visual Design

In total, 64 (80%) eHealth websites scored ≥3.5 on visual design (Multimedia Appendix 3). These eHealth websites were assessed as having an attractive visual design, an appealing color scheme, were well structured with a consistent layout, and the content was easy to read. They also displayed appropriately sized fonts, buttons, and menus. Some examples include Pelvic Pain-Pain Australia [34], Endometriosis-Healthline [35], and Endometriosis Practice Essentials-Medscape [36].

Of the 64 eHealth websites that scored well on visual design, 35 (54%) provided education on natal female pain to the endometriosis community, 22 (34%) were business pages of organizations, 4 (6%) provided education to health care providers on the management of conditions that cause natal female pain, and 2 (3%) were related to endometriosis research.

#### User Engagement

A low number of eHealth websites (n=18, 23%) scored highly (≥3.5) on user engagement (Multimedia Appendix 3). Websites that scored highly were characterized by a good mix of text, images, and videos. The content was presented interactively and engagingly. User engagement was further enhanced by avoiding features like pop-up ads, notifications, alerts, and sounds. Some examples include Adenomyosis—Sydney Morning Herald [37], Adenomyosis—The Centre for Innovative Gyn Care [38], and Adenomyosis—The Medical Republic [31]. Of the 18 eHealth websites that scored well on user engagement, 11 (61%) provided education on natal female pain to the community, 5 (28%) were business pages of private organizations, and 2 (11%) provided education to health care providers.

#### Content

A total of 63 (79%) eHealth websites scored well (≥3.5) on the content domain (Multimedia Appendix 3). These eHealth websites contained appropriate, complete, and concise information with clarity about the eHealth website’s purpose. However, of 63 websites, only 40% (n=25) of the eHealth websites provided references or mentioned the sources of information, and only 38% (n=26) provided the name of the author. Examples of websites that scored highly for content included Endometriosis—Jean Hailes for Women’s Health [33], NewsGP—RACGP [39], and Endometriosis—Better Health Channel [40].

Thirty-five (56%) of these eHealth websites provided education on natal female pain to the endometriosis community, 17 (27%) were business pages of private organizations, 5 (8%) provided education to health care providers, 5 (8%) were related to endometriosis research, and 1 (2%) was a media release article on endometriosis by an Australian University.

#### Therapeutic Persuasiveness

Only 2 (2%) eHealth websites (ie, Endometriosis Australia Facebook Page [29] and Endometriosis UK [30]) scored highly (≥3.5) on the therapeutic persuasiveness domain (Multimedia Appendix 3). One page [29] is designed for the endometriosis community on Facebook, while the other page [30] is the official website of a United Kingdom–based endometriosis charitable organization. Both websites provide resources to raise awareness and educate people about endometriosis. These pages provide opportunities for a call to action, which can be described as activities that prompt the user to take action (eg, goal setting). Both websites facilitate interactions between the digital endometriosis community, release information about upcoming events, and enable engagement with page content such as watching informative videos [29] or engaging in a web chat [30].

#### Therapeutic Alliance

Only 28% (n=22) of the eHealth websites scored highly (≥3.5) on the therapeutic alliance domain (Multimedia Appendix 3). Seventeen (77%) eHealth websites provide education on natal female pain to the endometriosis community, 3 (14%) were business pages of private organizations, 1 (5%) provided education to health care providers, and 1 (5%) was related to endometriosis research. These eHealth websites incorporated features that sought to foster a therapeutic alliance with the user. Examples of support include personal stories of people affected by endometriosis, which creates a sense of a shared digital endometriosis community, support group information, and helpline numbers. Examples include Endometriosis UK [30], Endometriosis—Jean Hailes for Women’s Health [33], Endometriosis Australia [41] and Pelvic Pain Foundation of Australia [42].

#### General Subjective Evaluation of eHealth Websites

This criterion evaluates the eHealth website’s potential to benefit users based on reviewers’ subjective scores. Only 38% (n=30) of eHealth websites scored highly (≥3.5) under this criterion (Multimedia Appendix 3). Nineteen (63%) eHealth websites provided education on natal female pain to the endometriosis community, 4 (13%) provided education to health care providers, 6 (20%) were business pages of private organizations, and 1 (3%) was a journal article.

## Discussion

### Summary

We conducted a comprehensive, multidimensional quality assessment of endometriosis eHealth websites using the ENLIGHT tool that captures quality constructs like persuasive design and therapeutic alliance, which are considered central to the successful uptake of eHealth websites among end users [22]. Our systematic review identified 4 high-quality endometriosis eHealth websites that can be used as educational resources for the community and health care providers. This is the first systematic review to use the ENLIGHT tool and comprehensively assess endometriosis eHealth websites.

### Principal Results

#### Quality of Endometriosis eHealth Websites

##### Overview

The proliferation of incorrect digital health information is a major concern [43]. A quality assessment of eHealth websites in the United States revealed that only 58% (n=58) met the criteria for accuracy and credibility of content [44]. In a systematic review by Hirsch et al [10] that included 54 eHealth websites providing information on endometriosis, over a third did not cite authorship and almost a half did not report references or sources of information.

##### Recommendation 1

The need for the development of accurate and evidence-based endometriosis eHealth websites for the community. We found similar results in our assessment indicating the need for eHealth websites to integrate information such as referencing and authorship to provide credibility. Furthermore, we found that eHealth websites that provide education to health care providers scored better than those that provided education to the community. Therefore, to reduce the proliferation of incorrect information, improved referencing and authorship on community-targeted eHealth websites will improve credibility.

Furthermore, some evidence in the literature states that the quality of endometriosis-related information on the internet centers around content that can be inaccurate and misleading [10]; however, there is little evidence to describe what the quality of information means to the endometriosis community. To some, quality centers around improving self-awareness about endometriosis to help make informed decisions [45]. While credible evidence-based content is a significant part of quality, factors that facilitate user engagement, therapeutic persuasiveness, and therapeutic alliance are also worthy of quality assessment for information obtained over the internet since these factors ensure successful and long-term uptake of eHealth websites [46].

#### Need to Enhance User Engagement, Therapeutic Persuasiveness, and Therapeutic Alliance

##### Overview

Most of the eHealth websites scored well (≥3.5) on usability (n=76, 95%) and visual design (n=64, 80%). Usability and visual design influence the user’s first impression and subsequent uptake and use [47,48]. However, we found a low percentage of eHealth websites scored well on user engagement (n=18, 23%). Current endometriosis eHealth websites are primarily informative and lack user interaction. A Cochrane review found that health platforms with interactive features have positive effects on users (improved knowledge, self-efficacy, behavior, and clinical outcomes) as compared to nonusers [49].

##### Recommendation 2

User engagement can be improved by providing interactive features that enable users to input and receive a reaction by providing personalized feedback. For example, the web chat feature on the Endometriosis UK website [30] allows the user to make an enquiry and receive feedback. User engagement is also strengthened by sharing lived experiences and stories and cocreating content that is meaningful to users, such as clinicians sharing clinical insights.

Therapeutic persuasiveness and therapeutic alliance could further enhance user engagement [22,46]. However, we found few eHealth websites that scored well on therapeutic persuasiveness (n=2, 3%) and therapeutic alliance (n=22, 28%). Therapeutic persuasiveness is positively correlated with real-world usage of eHealth websites, while therapeutic alliance enhances positive user engagement by fostering relatability [46].

##### Recommendation 3

Since most endometriosis eHealth websites are informative only, therapeutic persuasiveness can be increased by adding a “call to action.” This means the eHealth website could suggest when to see a general practitioner, what to discuss at the medical appointment, or provide evidence-based self-management strategies to cope with endometriosis. Additionally, eHealth websites can incorporate conversational agents or chatbots such as Alexa (Google), Siri (Apple), S Voice or Bixby (Samsung), and Cortana (Microsoft Corp) [46,50], which mimic human conversations [46,50] to foster relatability. Therapeutic persuasiveness and therapeutic alliance can be further strengthened by sharing lived experiences through digital community engagement and cocreating meaningful content.

#### Evidence-Based Insights on Optimization of eHealth Websites

##### Overview

Our Google search for “endometriosis” identified a mix of eHealth websites with eclectic primary purposes including education, marketing for business, and dissemination of academic research. During internet searches, people are most likely to click on the first 5 websites that come up on the Google search result pages [51]. To attract traffic, an eHealth website should appear in the first 5 rankings [51].

##### Recommendation 4

Organizations could benefit from investing in search engine optimization (SEO) tools. SEO improves the ranking of a website in Google search results. There are various tools to achieve this including the creation of fresh, unique, and qualitative content [52] and improving keyword density, which is the amount of time the keywords that the users are searching for appear on the eHealth website. Including keywords in the headers, main titles, and content of the eHealth website also improves search ranking [51,52]. The use of meta descriptions, a short description (160 characters) of the website’s content that appears below the page title on the search result page and is managed by the website owner, can also improve ranking. The meta descriptions should include words that the target audience are likely to search for [51]. The use of permalinks (permanent and specific URL links) improves SEO. Proper link architecture helps the search engine discover the eHealth website [51,52]. Adding backlinks (other websites linking back to the main eHealth website) increases discoverability and is an important factor in improving rankings [51,52]. Social media promotions on popular platforms such as Facebook, Instagram, and Twitter help to popularize the content of the eHealth website, thereby improving a website’s ranking [51,52].

Other tools that can improve an eHealth website’s ranking include submitting the sitemap (ie, a list of pages, videos, and other content on the website and the relationship between them) to Google. This helps Google to download and index information. Google analyses this information to produce search results [53].

#### Endometriosis eHealth Websites as Resources for Health Care Providers

##### Overview

Our systematic review found only 1 high-quality eHealth website for health care providers Adenomyosis—The Medical Republic [31], compared to 3 for the endometriosis community (Table 2), indicating a lack of high-quality eHealth websites for health care providers. Evidence suggests that health care providers benefit from using eHealth websites in daily practice [54]. However, barriers such as difficulty accessing full-text documents, subscription fees, concerns about quality, and limited relevance of the information in day-to-day clinical practice may limit use [13]. Coupled with an identified need to improve endometriosis education among health care providers [4], it is necessary to develop digital learning tools to address this gap.

##### Recommendation 5

There is a need to develop easily accessible, evidence-based endometriosis eHealth websites for health care providers that provide a valuable and easy reference in daily practice and enhance professional development for endometriosis management.

### Strengths

Our systematic review has several strengths. Novel findings are presented on high-quality eHealth websites assessed using the ENLIGHT quality assessment tool and guide the community and health care providers toward quality, credible, and supportive health information. Health care providers can use these eHealth websites as educational resources and recommend high-quality eHealth websites to their patients. Finally, this review presents recommendations (ie, good design features and use of SEO tools) when designing or updating endometriosis eHealth websites as evidence suggests that good design features can help improve a website’s ranking, reach, and discoverability [52].

### Limitations

Our study has the following limitations. The ENLIGHT quality assessment criteria [22] were challenging to apply to websites. Most eHealth websites did not provide a health or behavior-related intervention for natal female pain. Hence, it was difficult to evaluate therapeutic persuasiveness and therapeutic alliance criteria in their entirety. We did not evaluate the entire eHealth website. We only evaluated the landing page or article the Google search engine result produced to mimic real-world circumstances. However, in some cases, this led to an uploaded document (the National Action Plan for Endometriosis on the EndoActive website) rather than the actual website, so it was not truly assessing an eHealth website. Google accounts for 92.26% of the global market share as compared to Microsoft Bing (2.83%) [20]. Additionally, Yahoo Search provides results generated by Microsoft Bing [21]. Hence, we reported results based on Google search only. Although, we searched in “incognito mode,” the Google algorithm may have automatically incorporated our location when searching, which may have influenced the results. Furthermore, the digital world is changing rapidly. The time and geographical location of the search may influence results conducted today versus the results presented above. Due to the lack of translation services, we did not include eHealth websites in languages other than English. We did not assess if the eHealth websites included in this study were developed using a genuine cocreation process hence, we do not know whether they are representative of all ethnicities or races. Finally, while we recommend the top 4 high-quality eHealth websites based on the ENLIGHT scoring system as good sources of information on endometriosis, we believe that the word “good” may well be influenced in context, that is, the generalizability of interpretation of good may vary for users in low income versus high-income countries. Nevertheless, this study has shown how eHealth websites can be assessed using the ENLIGHT checklist, which is a validated tool with its 7 quality assessment criteria.

### Conclusions

Our systematic review presents novel findings on the quality assessment of eHealth websites using the ENLIGHT checklist to obtain endometriosis-related information. The findings of our study are (1) suggestive of high-quality eHealth websites for community use and (2) can be used by health care providers for educational purposes and recommendations. We recommend the development of (1) accurate and evidence-based endometriosis eHealth websites for the community; (2) accessible endometriosis eHealth websites for health care providers supporting daily practice and professional development; (3) interactive eHealth websites that promote user engagement, therapeutic persuasiveness, and therapeutic alliance; and (4) leveraging SEO tools to improve Google search ranking.

## Appendix

Multimedia Appendix 1 PRISMA (Preferred Reporting Items for Systematic Reviews and Meta-Analyses) 2020 checklist and PRISMA 2020 abstracts checklist.

Multimedia Appendix 2 Details of eHealth websites included in this study (listed alphabetically).

Multimedia Appendix 3 ENLIGHT quality assessment total score.

## Abbreviations

PRISMA: Preferred Reporting Items for Systematic Reviews and Meta-Analyses
RACGP: Royal Australian College of General Physicians
SEO: search engine optimization

## Data Availability

All data generated or analyzed during this study are included in this published article and Multimedia Appendices 1-3.
