# Supplementary material for: High-Quality eHealth Websites for Information on Endometriosis: Systematic Search
Source: J Med Internet Res. 2024 Jan 10;26:e48243. doi: 10.2196/48243 (PMC10809066; doi:10.2196/48243)
Supplement: Multimedia Appendix 2 [file jmir_v26i1e48243_app2.docx]

**Table S1. Details of eHealth websites included**

| **Name of the e-health website / Available on / URL** | **Developer** | **Funder** | **Intended purpose** | **Target audience** | **Category** | **Functionality** | **Country of origin** | **Last updated** |
| --- | --- | --- | --- | --- | --- | --- | --- | --- |
| 16 endometriosis symptoms – medicine net  [16 Endometriosis Symptoms: Treatment, Causes, 4 Stages, Surgery & Diagnosis (medicinenet.com)](https://www.medicinenet.com/endometriosis/article.htm) | Medicine Net Inc (WebMD LLC) | Not available | Provides information on what is endometriosis, signs and symptoms and management of endometriosis | People affected with endometriosis / anyone who wants to know about endometriosis | Health media organisation | Education | United States of America | June 2020 |
| A new solution to women's severe pain that isn't a hysterectomy  [A new solution to women's severe pain that isn't a hysterectomy (smh.com.au)](https://www.smh.com.au/national/a-new-solution-to-women-s-severe-pain-that-isn-t-a-hysterectomy-20181001-p5072s.html) | Sydney Morning Herald | Not available | News article on information about adenomyosis and uterine artery embolisation (procedure done to alleviate symptoms of adenomyosis) | People affected with adenomyosis / anyone who wants to know about adenomyosis | Health news article | Education | Australia | October 2018 |
| About Endo - Endometriosis Australia  [Endometriosis Australia \| About Endo](https://www.endometriosisaustralia.org/about-endo) | Endometriosis Australia | Not available | Provides information about endometriosis, signs and symptoms, diagnosis, treatment, fertility. Provides factsheets and FAQs on endometriosis | People affected with endometriosis / anyone who wants to know about endometriosis | Not-for-profit organisation | Education and support | Australia | Not available |
| Adenomyosis  [Adenomyosis - Endo Health](https://endohealth.com.au/gynaecological-conditions/adenomyosis/) | Endo Health | Not available | Provides information on what is adenomyosis, symptoms, causes, risk factors, diagnosis and treatment | People affected with adenomyosis / anyone who wants to know about adenomyosis | Private organisation | Business page | Australia | Not available |
| Adenomyosis  [Adenomyosis: Causes, Symptoms, Diagnosis & Treatment (clevelandclinic.org)](https://my.clevelandclinic.org/health/diseases/14167-adenomyosis) | Cleveland Clinic | Not available | Provides information on adenomyosis, symptoms, diagnosis, and treatment.  Informative appointment booking website of Cleveland Clinic | People affected with adenomyosis / anyone who wants to know about adenomyosis | Private organisation | Business page | United States of America | Not available |
| Adenomyosis  [Adenomyosis (nhsinform.scot)](https://www.nhsinform.scot/healthy-living/womens-health/girls-and-young-women-puberty-to-around-25/periods-and-menstrual-health/adenomyosis) | NHS Inform Scotland | The UK Government | Provides information on adenomyosis, symptoms, diagnosis, treatment and where to get help | People affected with adenomyosis / anyone who wants to know about adenomyosis | Government department NHS Inform Scotland | Education | United Kingdom | February 2023 |
| Adenomyosis  Adenomyosis - NHS (www.nhs.uk) | National Health Service | The UK Government | Provide information on the overview, treatment and complications of adenomyosis. | People affected with adenomyosis / anyone who wants to know about adenomyosis | Government department | Education | United Kingdom | July 2023 |
| Adenomyosis  [Uterine Adenomyosis > Fact Sheets > Yale Medicine](https://www.yalemedicine.org/conditions/uterine-adenomyosis) | Yale Medicine | Not available | Provides general information about adenomyosis and supports appointment booking. | People affected with adenomyosis/ anyone who wants to know about adenomyosis | University | Business page | United States of America | Not available |
| Adenomyosis  [Adenomyosis \| Pelvic Pain Support Network](https://www.pelvicpain.org.uk/conditions/adenomyosis/) | Pelvic Pain Support Network | Not available | Provides information on adenomyosis, symptoms, diagnosis, and treatment | People affected with adenomyosis/ anyone who wants to know about adenomyosis | Not-for-profit organisation | Education and Support | United Kingdom | Not available |
| Adenomyosis  [Adenomyosis \| Temple Health](https://www.templehealth.org/services/conditions/adenomyosis) | Temple Health | Not available | Provides information on adenomyosis, symptoms, diagnosis, and treatment. Informative appointment booking website of Temple Health | People affected with adenomyosis/ anyone who wants to know about adenomyosis | Private organisation | Business page | United States of America | Not available |
| Adenomyosis  [Adenomyosis \| Northern Beaches Interventional Radiology (nbir.com.au)](https://www.nbir.com.au/adenomyosis) | Northern Beaches Interventional Radiology | Not available | Provides information on adenomyosis, symptoms, diagnosis, and treatment. Informative appointment booking website of Northern Beaches Interventional Radiology | People affected with adenomyosis/ anyone who wants to know about adenomyosis | Private organisation | Business page | Australia | Not available |
| Adenomyosis  [Adenomyosis \| HonorHealth](https://www.honorhealth.com/medical-services/gynecology/gynecology-symptoms-conditions/adenomyosis) | Honor Health | Not available | Provides information on adenomyosis, symptoms, diagnosis, and treatment. Informative appointment booking website of Honor Health | People affected with adenomyosis/ anyone who wants to know about adenomyosis | Private organisation | Business page | United States of America | Not available |
| Adenomyosis  [ADENOMYOSIS - Brisbane Centre for Endometriosis](https://brisbanecentreforendometriosis.com.au/2021/11/26/adenomyosis/) | Brisbane Centre for Endometriosis | Not available | Provides information on adenomyosis, symptoms, diagnosis, and treatment. Informative appointment booking website of Brisbane Centre for Endometriosis | People affected with adenomyosis/ anyone who wants to know about adenomyosis | Private organisation | Business page | Australia | Not available |
| Adenomyosis  <https://emedicine.medscape.com/article/2500101-overview?form=fpf> | Medscape (WebMD LLC) | Not available | Provides information on adenomyosis for healthcare practitioners | Healthcare practitioners | Health media organisation | Education for healthcare professionals | United States of America | June 2023 |
| Adenomyosis –  [Adenomyosis: Symptoms, Diagnosis, Treatment, and More (healthline.com)](https://www.healthline.com/health/adenomyosis) | Healthline | Not available | Health information website that provides information on symptoms, treatments, causes, diagnosis, complications and risk factors of adenomyosis | People affected with adenomyosis / anyone who wants to know about adenomyosis | Health media organisation | Education | United States of America | November 2021 |
| Adenomyosis - [Adenomyosis \| Johns Hopkins Medicine](https://www.hopkinsmedicine.org/health/conditions-and-diseases/adenomyosis) | Johns Hopkins University, The Johns Hopkins Hospital, and Johns Hopkins Health System | Not available | Provides general information about adenomyosis and supports appointment booking | People affected with adenomyosis/ anyone who wants to know about adenomyosis | University | Business page | United States of America | Not available |
| Adenomyosis – CARE  <https://www.sydneycare.com.au/patients/conditions/adenomyosis/> | CARE (Centre for Advanced Reproductive Endosurgery | Not available | Provides information about adenomyosis, signs and symptoms, diagnosis, treatment | People affected with adenomyosis / anyone who wants to know about adenomyosis | Private organisation | Business page | Australia | Not available |
| Adenomyosis – Guys and St Thomas – NHS  <https://www.guysandstthomas.nhs.uk/resources/patient-information/gynaecology/adenomyosis.pdf> | Guy's and St Thomas NHS Foundation Trust | Not available | Informative leaflet (PDF) on adenomyosis and where to get help | People affected with adenomyosis / anyone who wants to know about adenomyosis | Government department (NHS) | Education | United Kingdom | January 2020 |
| Adenomyosis - Healthdirect Australia  [Adenomyosis \| healthdirect](https://www.healthdirect.gov.au/adenomyosis) | Australian Govt - Department of health | Not available | Provides information provides information on what is endometriosis, causes, symptoms, diagnosis and treatment of adenomyosis | People affected with endometriosis / researchers / anyone who wants to know about adenomyosis | Government department | Education | Australia | Not available |
| Adenomyosis – Jean Hailes /  <https://www.jeanhailes.org.au/health-a-z/vulva-vagina-ovaries-uterus/adenomyosis> | Jean Hailes for Women’s Health | Australian Government | Informative, runs women's health clinics in Victoria. Provides information on causes, treatment, diagnosis of adenomyosis and when to seek help | People affected with adenomyosis / anyone who wants to know about adenomyosis | Not-for-profit organisation | Education and support | Australia | March 2020 |
| Adenomyosis – sister to endometriosis or distant cousin /  <https://www.endometriosisaustralia.org/post/2016/11/08/adenomyosis-sister-to-endometriosis-or-distant-cousin> | Endometriosis Australia | Not available | Informative article on adenomyosis - causes, symptoms, diagnosis, treatment and complications of adenomyosis | People affected with adenomyosis / anyone who wants to know about adenomyosis | Not-for-profit organisation | Education | Australia | Not available |
| Adenomyosis – symptoms, causes and treatment – Medical News July /  <https://www.medicalnewstoday.com/articles/321296> | Medical News Today (Part of Healthline Media, UK) | Not available | Provides information on symptoms, treatments, causes, diagnosis, complications and risk factors of adenomyosis, when to see a doctor and fertility and pregnancy management | People affected with adenomyosis / anyone who wants to know about adenomyosis | Health media organisation | Education | United Kingdom | Not available |
| Adenomyosis – symptoms, causes and treatments – Innovative Gyn /  <https://innovativegyn.com/conditions/adenomyosis> | Centre for Innovative GYN care | Not available | Provides information on introduction, symptoms, causes, diagnosis, treatment, consultation facility for adenomyosis. | People affected with adenomyosis / anyone who wants to know about adenomyosis | Private organisation | Business page | United States of America | Not available |
| Adenomyosis – Wikipedia  [Adenomyosis - Wikipedia](https://en.wikipedia.org/wiki/Adenomyosis) | Wikipedia - online free content encyclopedia | Not available | Provides information on adenomyosis, causes, signs and symptoms, mechanism diagnosis, treatment and prognosis | People affected with adenomyosis / anyone who wants to know about adenomyosis | Encyclopedia | Education | United States of America | July 2020 |
| Adenomyosis Epidemiology, Risk Factors, Clinical Phenotype and Surgical and Interventional Alternatives to Hysterectomy  [Adenomyosis: Epidemiology, Risk Factors, Clinical Phenotype and Surgical and Interventional Alternatives to Hysterectomy - PMC (nih.gov)](https://www.ncbi.nlm.nih.gov/pmc/articles/PMC3859152/) | Geburtshilfe Frauenheilkd (German Society of Gynecology and Obstetrics) | Not available | Journal article on adenomyosis epidemiology, risk factors, clinical phenotype and surgical and interventional alternatives to hysterectomy. | People affected with adenomyosis / anyone who wants to know about adenomyosis / academicians / researchers | Journal article | Research | International | Not applicable |
| Adenomyosis symptoms and causes - Mayo Clinic / <https://www.mayoclinic.org/diseases-conditions/adenomyosis/symptoms-causes/syc-20369138#:~:text=Sometimes%2C%20adenomyosis%20causes%20no%20signs,pelvic%20pain%20during%20menstruation%20(dysmenorrhea)> | Mayo Clinic | Not available | Provides information on adenomyosis, causes, signs and symptoms, diagnosis, and treatment.  Informative, appointment booking website for Mayo Clinic | People affected with adenomyosis / anyone who wants to know about adenomyosis | Private organisation | Business page | United States of America | Not available |
| Adenomyosis: symptoms, causes and treatments -Web MD /  <https://www.webmd.com/women/guide/adenomyosis-symptoms-causes-treatments> | WebMD LLC | Not available | Health information website. Provides information on what is adenomyosis, symptoms, risk factors, diagnosis, treatment and prognosis | People affected with adenomyosis / anyone who wants to know about adenomyosis | Health media organisation | Education | United States of America | Not available |
| Adenomyosis: The poor cousin of endometriosis • The Medical Republic  [Adenomyosis: The poor cousin of endometriosis • The Medical Republic](https://medicalrepublic.com.au/adenomyosis-the-poor-cousin-of-endometriosis/25274) | The Medical Republic | Not available | News article - on what is adenomyosis, causes, symptoms, diagnosis, treatment, complications, impact on fertility and pregnancy | Primarily a news website for healthcare providers  People affected with adenomyosis | Health media organisation | Education | Australia | Not available |
| Australia’s first endometriosis and pelvic pain clinics now available nationally - Australian Government Department of Health and Aged Care  [Australia’s first endometriosis and pelvic pain clinics now available nationally \| Health Portfolio Ministers \| Australian Government Department of Health and Aged Care](https://www.health.gov.au/ministers/the-hon-ged-kearney-mp/media/australias-first-endometriosis-and-pelvic-pain-clinics-now-available-nationally#:~:text=Endometriosis%20affects%20at%20least%201,of%20seven%20years%20before%20diagnosis.) | Australian Government Department of Health and Aged Care | Australian Government | Provides information on Australia’s first endometriosis and pelvic pain clinics | People affected with endometriosis / anyone who wants to know about endometriosis and actions taken by the Australian Government | Government department | Education | Australia | March 2023 |
| Could I have endometriosis?  [Could I have endometriosis? Signs, symptoms and what to do next \| HCF](https://www.hcf.com.au/health-agenda/women/endometriosis/symptoms) | HCF | Not available | Provides information on endometriosis, symptoms, diagnosis and treatment | People affected with endometriosis / anyone who wants to know about endometriosis | Private organisation | Business page | Australia | Not available |
| Difference between endometriosis and adenomyosis  <https://www.circlehealthgroup.co.uk/health-matters/womens-health/the-difference-between-ednometriosis-and-adenomyosis> | Circle Health |  | Provides information on the difference between endometriosis and adenomyosis | People affected with endometriosis and/or adenomyosis / anyone who wants to know about endometriosis /adenomyosis | Private organisation | Business page | United Kingdom | Not available |
| Endometriosis  [Endometriosis - Symptoms and causes - Mayo Clinic](https://www.mayoclinic.org/diseases-conditions/endometriosis/symptoms-causes/syc-20354656) | Mayo Clinic | Not available | Provides information on endometriosis, symptoms, diagnosis and treatment.  Informative appointment booking website of Mayo Clinic | People affected with endometriosis / anyone who wants to know about endometriosis | Private organisation | Business page | United States of America | Not available |
| Endometriosis  [Endometriosis (who.int)](https://www.who.int/news-room/fact-sheets/detail/endometriosis) | World Health Organisation (WHO) | Not available | Provides information on endometriosis, symptoms, diagnosis, treatment and WHO response. | People affected with endometriosis / anyone who wants to know about endometriosis | Not-for-profit organisation | Education | International | March 2023 |
| Endometriosis - Better Health Channel  [Endometriosis - Better Health Channel](https://www.betterhealth.vic.gov.au/health/conditionsandtreatments/endometriosis) | Department of Health & Human Services, State Government of Victoria, Australia | Not available | Provides information on what is endometriosis, causes, symptoms, diagnosis and treatment of endometriosis, alternative medicines and where to get help | People affected with endometriosis / anyone who wants to know about endometriosis | Health media organisation | Education | Australia | March 2018 |
| Endometriosis - Healthdirect Australia  [Endometriosis \| healthdirect](https://www.healthdirect.gov.au/endometriosis) | Australian Govt - Department of health | Not available | Provides information provides information on what is endometriosis, causes, symptoms, diagnosis and treatment of endometriosis | People affected with endometriosis / researchers / anyone who wants to know about endometriosis | Government department | Education | Australia | Not available |
| Endometriosis - IVF Australia  [Endometriosis \| Symptoms, Causes & Fertility \| Surgery \| IVF Australia](https://www.ivf.com.au/planning-for-pregnancy/female-fertility/endometriosis) | IVF Australia | Not available | Information on what is endometriosis, symptoms, causes, diagnosis, treatment of endometriosis, how does it affect fertility. Provides a portal to book appointments | People affected with endometriosis / anyone who wants to know about endometriosis | Private organisation (Business page) | Business page | Australia | Not available |
| Endometriosis - Jean Hailes  [Endometriosis \| Jean Hailes](https://www.jeanhailes.org.au/health-a-z/endometriosis) | Jean's Health for Women's Health | Not available | The organisation runs women's health clinics in Victoria. Provides information on causes, treatment, diagnosis, complications, emotions, fertility and sex issues associated with endometriosis and resources for endometriosis | People affected with endometriosis / researchers / anyone who wants to know about endometriosis | Not-for-profit organisation | Education and support | Australia | Not available |
| Endometriosis – Johns Hopkins medicine /  <https://www.hopkinsmedicine.org/health/conditions-and-diseases/endometriosis> | Johns Hopkins University, The Johns Hopkins Hospital, and Johns Hopkins Health System | Not available | Provides general information about endometriosis and supports appointment booking | People affected with endometriosis / anyone who wants to know about endometriosis | University | Business page | United States of America | Not available |
| Endometriosis – NHS /  <https://www.nhs.uk/conditions/endometriosis/> | National Health Service | The UK Government | Provide information on the overview, treatment and complications of endometriosis | People affected with endometriosis / anyone who wants to know about endometriosis | Government department | Education | United Kingdom | January 2019 |
| Endometriosis – O&G Magazine  [Endometriosis – O&G Magazine (ogmagazine.org.au)](https://www.ogmagazine.org.au/16/2-16/endometriosis/) | O&G magazine run by RANZCOG | Not available | Provides information on treatment of endometriosis | People affected with endometriosis / anyone who wants to know about endometriosis | Professional organisation | Education for healthcare professionals | Australia | Last updated 6 years ago |
| Endometriosis – practice essentials  [Endometriosis: Practice Essentials, Background, Pathophysiology (medscape.com)](https://emedicine.medscape.com/article/271899-overview) | Medscape (WebMD LLC) | Not available | Provides information on endometriosis for healthcare practitioners | Healthcare practitioners | Health media organisation | Education for healthcare professionals | United States of America | July 2018 |
| Endometriosis - SHINE SA  [Endometriosis - SHINE SA](https://shinesa.org.au/health-information/endometriosis/endometriosis/) | Shine South Australia | Not available | Provides information on what is endometriosis, signs and symptoms, causes, diagnosis and treatment of endometriosis. | People affected with endometriosis / anyone who wants to know about endometriosis | Private organisation | Business page | Australia | September 2019 |
| Endometriosis – Wikipedia  <https://en.wikipedia.org/wiki/Endometriosis> | Wikipedia - online free content encyclopedia | Not available | Provides information on endometriosis, causes, signs and symptoms, mechanism diagnosis, treatment and prognosis | People affected with endometriosis / anyone who wants to know about endometriosis | Encyclopedia | Education | United States of America | August 2020 |
| Endometriosis – Women’s health.gov /  <https://www.womenshealth.gov/a-z-topics/endometriosis> | Office on Women's Health - US Department of Health and Human services | Not available | Provides information on what is endometriosis, symptoms, causes, diagnosis, treatment, fertility and other FAQs | People affected with endometriosis / anyone who wants to know about endometriosis | Government department | Education | United States of America | April 2019 |
| Endometriosis (for teens) – Nemours kids health  <https://kidshealth.org/en/teens/endometriosis.html> | Nemours (not-for-profit children's health system) | Not available | Informative - Provides general information on endometriosis and provides an online portal for appointment booking for management of endometriosis | People including teenagers affected with endometriosis / anyone who wants to know about endometriosis | Private organisation | Business page | United States of America | February 2014 |
| Endometriosis and Adenomyosis  [Endometriosis & Adenomyosis \| Women's and Men's Health Physiotherapy (wmhp.com.au)](https://www.wmhp.com.au/womens-pelvic-health/endometriosis-and-adenomyosis) | Women's and Men's Health Physiotherapy | Not available | Provides information on endometriosis and adenomyosis and the how pelvic physiotherapy can help. Informative appointment booking website of Women's and Men's Health Physiotherapy | People affected with pelvic pain and looking for information on pelvic physiotherapy. | Private organisation | Business page | Australia | Not available |
| Endometriosis Australia\| Facebook Page  [(3) Endometriosis Australia \| Facebook](https://www.facebook.com/EndometriosisAustralia/) | Endometriosis Australia | Not available | The forum is meant for discussion, to raise awareness and educate people about endometriosis | People affected with endometriosis / anyone who wants to know about endometriosis | Not-for-profit organisation | Education and support | Australia | Not applicable |
| Endometriosis Care Centre Australia: ECCA  [ECCA – Endometriosis Care Centre Australia](https://ecca.com.au/) | Endometriosis Care Centre of Australia | Not available | Informative – Provides information on what is endometriosis, symptoms, causes, diagnosis, treatment of endometriosis | People affected with endometriosis / anyone who wants to know about endometriosis | Private organisation | Business page | Australia | Not available |
| Endometriosis causes, complications and treatment Health line / <https://www.healthline.com/health/endometriosis> | Healthline | Not available | Health information website - to seek information on symptoms, treatments, causes, diagnosis, complications and risk factors of endometriosis | People affected with endometriosis / anyone who wants to know about endometriosis | Health media organisation | Education | United States of America | Not available |
| Endometriosis costs women and society $30,000 a year for every sufferer  [Endometriosis costs women and society $30,000 a year for every sufferer (theconversation.com)](https://theconversation.com/endometriosis-costs-women-and-society-30-000-a-year-for-every-sufferer-124975) | The Conversation – Media Group | Not available | News article – provides information on the prevalence and cost of endometriosis | People affected with endometriosis / researchers / academicians | Health media organisation | Education | Australia | Not available |
| Endometriosis in Australia - Australian Institute of Health and Family Welfare  <https://www.aihw.gov.au/getmedia/a4ba101d-cd6d-4567-a44f-f825047187b8/aihw-phe-247.pdf.aspx?inline=true> | Australian Institute of Health and Family Welfare | Not available | Provides information on the prevalence and hospitalisation due to endometriosis in Australia. | People affected with endometriosis / researchers / anyone who wants to know about endometriosis | Government organisation | Education | Australia | August 2019 (published) |
| Endometriosis Symptoms & Treatment – WHRIA  [Endometriosis Symptoms & Treatment - WHRIA](https://www.whria.com.au/for-patients/pelvic-pain/endometriosis/) | Women's Health and Research Institute of Australia | Not available | Information on what is endometriosis, symptoms, causes, diagnosis, treatment of endometriosis, how does it affect fertility. Provides a portal to book appointments | People affected with endometriosis / anyone who wants to know about endometriosis | Private organisation | Business page | Australia | Not available |
| Endometriosis: Health system 'oblivious to suffering' of one in 10 women affected by silent epidemic  [Endometriosis: Health system 'oblivious to suffering' of one in 10 women affected by 'silent epidemic' - ABC News](https://www.abc.net.au/news/2017-03-24/endometriosis-health-system-oblivious-to-suffering-of-women/8331534) | ABC News | Not available | Blog article - provides information on burden of endometriosis | People affected with endometriosis / anyone who wants to know about endometriosis | Health news article | Education | Australia | Not available |
| Endometriosis: symptoms, diagnosis and treatment – clue /  <https://helloclue.com/articles/cycle-a-z/endometriosis-101> | Clue app | Not available | Clue is an app for period tracking and Clue has an online website that runs as an encyclopedia.  This page provides information on endometriosis 101 - causes, symptoms, diagnosis and management and tracking using the Clue app | People affected with endometriosis / anyone who wants to know about endometriosis | Private organisation | Business page | Not explicitly stated (?Germany) | Not available |
| Endometriosis: what is endometriosis – UCLA Health  [Endometriosis: What is Endometriosis? Endometriosis Symptoms, Treatment, Diagnosis - UCLA (uclahealth.org)](https://www.uclahealth.org/obgyn/endometriosis) | UCLA Health - Obsteterics and Gynaecology | Not available | Provides information about endometriosis. Provides a portal for appointment booking with UCLA doctors | People affected with endometriosis / anyone who wants to know about endometriosis | University | Business page | United States of America | Not available |
| Epworth Clinical Trials  [Current research studies for endometriosis in Australia \| Julia Argyrou Endometriosis Centre (epworth.org.au)](https://www.epworth.org.au/our-services/endometriosis-centre/clinical-trials) | Epworth Foundation | Not available | Endometriosis research related page providing information on research related to endometriosis | Researchers, clinicians, people who want to participate in endometriosis research | Not-for-profit organisation | Research | Australia | August 2023 |
| For Women – Pelvic Pain Foundation of Australia [For Women - Pelvic Pain Foundation](https://www.pelvicpain.org.au/learn/for-women/) | Pelvic Pain Foundation of Australia | Not available | Provides information on pelvic pain in women and those assigned female at birth | People affected with pelvic pain or anyone who wants to know about pelvic pain | Not-for-profit organisation | Education and support | Australia | Not available |
| How We Help - Pelvic Pain Foundation  [Pelvic Pain Foundation - Home](https://www.pelvicpain.org.au/) | Pelvic Pain Foundation of Australia (not-for-profit) | Not available | Provides information on causes and management of pelvic pain | People affected with pelvic pain or anyone who ants to know about pelvic pain | Not-for-profit organisation | Education and support | Australia | Not available |
| Introducing Adenomyosis -  [Introducing Adenomyosis — QENDO](https://www.qendo.org.au/blog/introducing-adenomyosis) | QENDO | Not available | Blog article that provides information on what is adenomyosis, symptoms, causes, risk factors, diagnosis and treatment | People affected with adenomyosis / anyone who wants to know about adenomyosis | Not-for-profit organisation | Education and support | Australia | April 2023 |
| Know the signs and symptoms of endometriosis – qld health /  <https://www.health.qld.gov.au/news-events/news/signs-symptoms-endometriosis> | Queensland government - Queensland Health | Not available | Provides information on what is endometriosis, signs and symptoms, management and when to seek help | People affected with endometriosis / anyone who wants to know about endometriosis | Government department | Education | Australia | June 2019 |
| Lessons from implementing the Australian National Action Plan for Endometriosis  [Lessons from implementing the Australian National Action Plan for Endometriosis - PMC (nih.gov)](https://www.ncbi.nlm.nih.gov/pmc/articles/PMC9346321/) | Reproduction and Fertility | Not available | Journal article on lessons learnt from implementing the Australian National Action Plan for Endometriosis in Australia | People affected with endometriosis, adenomyosis or chronic pelvic pain /researchers /policy makers. | Journal article | Research | International | Not applicable |
| National Action Plan for Endometriosis – EndoActive  [National Action Plan for Endometriosis (endoactive.org.au)](https://endoactive.org.au/wp-content/uploads/13.National-Action-Plan-for-Endometriosis.pdf) | Australian Government - Department of Health | Australian Government | Document detailing the National Action Plan for Endometriosis | People affected with endometriosis / researchers / clinicians / anyone who wants to know about endometriosis | Government Department | Education | Australia | July 2018 (published) |
| New hope for the 700,000 Australia women suffering from endometriosis  [New hope for the 700,000 Australia women suffering from endometriosis \| Clinical Knowledge Network (ckn.org.au)](https://www.ckn.org.au/content/new-hope-700000-australia-women-suffering-endometriosis) | Clinical Knowledge Network | Not available | Blog article that provides information on burden of endometriosis, new developments and signs and symptoms and government initiative through the National Action Plan for Endometriosis | Primarily for staff working in Queensland Public Health Sector.  People affected with endometriosis / researchers | Government department | Education | Australia | Not available |
| New pelvic pain and endometriosis clinic in Australia  [New pelvic pain and endometriosis clinics are opening across Australia. Where will they be? - ABC News](https://www.abc.net.au/news/2023-03-22/where-new-pelvic-pain-and-endometriosis-clinics-will-be/102126832) | ABC News | Not available | Blog article - provides information on burden of endometriosis | People affected with endometriosis / anyone who wants to know about endometriosis | Health news article | Education | Australia | March 2023 |
| One in nine Australian women aged 40–44 has endometriosis  [RACGP - One in nine Australian women aged 40–44 has endometriosis](https://www1.racgp.org.au/newsgp/clinical/one-in-nine-australian-women-diagnosed-with-endome) | NewsGP - News hub of RACGP | Not available | News article for healthcare providers - on the prevalence of endometriosis | Primarily for GPs /  People affected with endometriosis | Professional organisation | Education for healthcare providers | Australia | Not available |
| One in nine Australian women live with endometriosis  [One in nine Australian women live with endometriosis - School of Public Health - University of Queensland (uq.edu.au)](https://public-health.uq.edu.au/article/2019/08/one-nine-australian-women-live-endometriosis) | The University of Queensland - School of Public Health | Not available | News article - provides information on the research conducted on endometriosis - Australian Longitudinal Study on Women's Health | People affected by endometriosis / anyone who wants know about endometriosis / researchers | University | Media Release | Australia | August 2019 |
| Pelvic Pain – Pain Australia  <https://www.painaustralia.org.au/about-pain/forms-of-pain-2021/pelvic-pain-2021> | Pain Australia | Not available | Provides information on pelvic pain | People affected with pelvic pain / or anyone who wants to know about pelvic pain | Advocacy organisation | Education and support | Australia | Not available |
| Pelvic Pain — Pain Specialists Australia  [Pelvic Pain — Pain Specialists Australia](https://painspecialistsaustralia.com.au/pelvic-pain) | Pain Specialists Australia | Not available | Provides information on pelvic pain and various conditions that may cause pelvic pain, signs and symptoms, diagnosis and management of pelvic pain. Provides a portal for appointment booking | People affected with pelvic pain / or anyone who wants to know about pelvic pain | Not-for-profit organisation | Education and support | Australia | Not available |
| Pelvic pain: what’s causing your pelvic pain – onhealth  [Pelvic Pain : What's Causing Your Pelvic Pain? (onhealth.com)](https://www.onhealth.com/content/1/pelvic_pain_causes) | Onhealth.com (WebMD LLC | Not available | Health information website - Provides information on conditions that cause pelvic pain, diagnosis and management | People affected with pelvic pain / anyone who wants to know about pelvic pain | Health media organisation | Education | United States of America | April 2020 |
| Recent advances in understanding and managing adenomyosis – ncbi  /  <https://www.ncbi.nlm.nih.gov/pmc/articles/PMC6419978/> | US National Library of Medicine | Not available | Journal article on recent advances in understanding and managing adenomyosis | People affected with adenomyosis / anyone who wants to know about adenomyosis / academicians / researchers | Journal article | Research | International | Not applicable |
| Some facts about endo - Endometriosis Australia  [Endometriosis Australia](https://endometriosisaustralia.org/) | Endometriosis Australia | Not available | Provides information on endometriosis, symptoms, diagnosis and treatment | People affected with endometriosis / anyone who wants to know about endometriosis | Not-for-profit organisation | Education and support | Australia | Not available |
| Supporting workers with endometriosis in the workplace \| Safe Work Australia  [Supporting workers with endometriosis in the workplace \| Safe Work Australia](https://www.safeworkaustralia.gov.au/doc/supporting-workers-endometriosis-workplace) | Safe Work Australia | Not available | Provides information for employers for supporting people with endometriosis, information for workers and general information on endometriosis | People affected with endometriosis / employers / workers | Government department | Education | Australia | March 2020 |
| The cost of illness and economic burden of endometriosis and chronic pelvic pain in Australia: A national online survey  [The cost of illness and economic burden of endometriosis and chronic pelvic pain in Australia: A national online survey (plos.org)](https://journals.plos.org/plosone/article?id=10.1371/journal.pone.0223316) | PLOS ONE Journal | Not available | Journal article on the economic burden of endometriosis in Australia | People affected with pelvic pain and endometriosis / anyone who wants to know about endometriosis / academicians / researchers | Journal article | Research | Australia | Not applicable |
| The Difference Between Endometriosis and Adenomyosis  [The Difference Between Endometriosis and Adenomyosis (webmd.com)](https://www.webmd.com/women/endometriosis/women-endometriosis-vs-adenomyosis) | Medicine Net Inc (WebMD LLC) | Not available | Provides information on what the difference between endometriosis and adenomyosis | People affected with endometriosis and/or adenomyosis / anyone who wants to know about endometriosis | Health media organisation | Education | United States of America | September 2022 |
| Understanding endometriosis - Endometriosis UK  [Understanding Endometriosis \| Endometriosis UK (endometriosis-uk.org)](https://www.endometriosis-uk.org/understanding-endometriosis) | Endometriosis UK | Not available | Provides information on what is endometriosis. | People affected with endometriosis / anyone who wants to know about endometriosis | Not-for-profit organisation | Education and support | United Kingdom | Not available |
| Very bad period or pelvic pain is not necessarily normal -  [RACGP - ‘Very bad period or pelvic pain is not necessarily normal’](https://www1.racgp.org.au/newsgp/clinical/very-bad-period-or-pelvic-pain-is-not-necessarily) | NewsGP - News hub of RACGP | Not available | News article for healthcare providers - on management of endometriosis | Primarily for GPs /  People affected with endometriosis | Professional organisation | Education for healthcare providers | Australia | Not available |
| What adenomyosis and endometriosis have in common  [What adenomyosis and endometriosis have in common - Epworth HealthCare](https://www.epworth.org.au/blog/2022/adenomyosis-endometriosis#:~:text=In%20essence%2C%20endometriosis%20is%20always,cause%20the%20uterus%20to%20enlarge.&text=The%20symptoms%20of%20adenomyosis%20are,period%2C%20and%20heavy%20menstrual%20bleeding.) | Epworth Foundation | Not available | Provides information on what is common between endometriosis and adenomyosis | People affected with endometriosis and/or adenomyosis / anyone who wants to know about endometriosis and adenomyosis | Not-for-profit organisation | Research | Australia | April 2023 |
| What we're doing about endometriosis – Australian Government Department of Health  [What we’re doing about endometriosis \| Australian Government Department of Health](https://www.health.gov.au/health-topics/chronic-conditions/what-were-doing-about-chronic-conditions/what-were-doing-about-endometriosis) | Australian Government Department of health | Not available | Provides information on what is endometriosis and measures taken by the Australian Govt to improve health of those affected with endometriosis | People affected with endometriosis / anyone who wants to know about endometriosis and actions taken by the Australian Government | Government department | Education | Australia | June 2020 |
| What you need to know about adenomyosis  - Eve Health  [WhWomensat you need to know about adenomyosis - Eve Health](https://evehealth.com.au/what-you-need-to-know-about-adenomyosis/) | Eve Health | Not available | Provides information on what is adenomyosis, symptoms, causes, risk factors, diagnosis and treatment | People affected with adenomyosis / anyone who wants to know about adenomyosis | Private organisation | Business page | Australia | 2021 |
| You've probably heard of endometriosis –  [Adenomyosis symptoms: 'Appalling' lack of awareness around condition that affects up to one in three women (9news.com.au)](https://www.9news.com.au/national/adenomyosis-symptoms-bad-cousin-endometriosis-health-condition-impacts-even-more-women-lack-of-awareness/ffceeb4c-3308-480c-a0d7-82c829294cbc) | 9 News | Not available | Provides information on what is adenomyosis, symptoms, causes, risk factors, diagnosis and treatment | People affected with adenomyosis / anyone who wants to know about adenomyosis | Health news article | Education | Australia | May 2023 |
